# Ozone

**Published:** 1868-09

**Authors:** 


### Ozone.

Dr. H. Day, in a report on this article made to the St. Andrews Medical Graduates' Association, gives the conclusions at which he has arrived, the most important of which we subjoin:

There can be no escaping, at this moment, from the theory that ozone is a modified condition of oxygen, indeed, is oxygen plus force, which force is probably used in condensation—in other words, the power or capability of oxygen to combine with itself.

For the production of ozone in the laboratory, no method is so good as that accomplished by the aid of the induction coil. The production of ozone in the air, if it be there, is not yet in any way definitely understood.

The ordinary tests for ozone are imperfect, not because they will not prove the presence of ozone, but because they prove too much—that is to say, the presence of other bodies also common to the atmosphere.

In its action on the body the effects of ozone seem to be confined to the respiratory passages and structures; in fact, it is purely local in its action, resembling closely diluted chlorine and diluted bromine in vapor; the phenomena induced, varying in intensity, may be catarrhal, bronchial, or pneumonic, nor is there any evidence of any other class of diseases from ozone.

On dead matter, ozone exerts a powerful destructive action, resembling in this way chlorine, iodine, and especially bromine.

Ozone is a disinfectant and deodorizer belonging to those bodies which disinfect and deodorize by resolving and decomposing into primitive and inocuous forms, competing in this respect with substances already named—*i. e.*, chlorine, bromine, and iodine. It possesses these qualities in a less degree than chlorine and bromine, and is, in many cases, not so applicable as iodine.

As a preventive of disease, ozone can only act by destroying organic animal poisons, in which respect it may be compared with the substances I have more than once named. With regard to the disinfecting and deodorizing powers of ozone, I would refer you to the opinions of the late Dr. Baker, contained in the Hastings prize essay for 1865. The subject of comparison, and indeed the whole subject of deodorizing and disinfecting, is there so admirably, so exhaustively discussed, as to leave, it seems to me, nothing further to be said on the subject.

Lastly, as a remedy. In the form of ozonized oil, of ozonized ether, and ozonized water, it once more ranks with a similar combination of remedies, containing chlorine, bromine, and especially iodine. Whether, in any respect, it may prove to have greater advantages than the last named trusty and ready agent, can only be conclusively arrived at by determining whether it will do what iodine will *not* do, and this can only be decisively made out by applying to it the test of inductive philosophy—a rigid exclusion of all that is ineffective.—*Amer. Journ. Med. Science, July,* 1868. *Med. Press and Circular, Jan.* 15, 1868.—*Journ. of Pharmacy.*

